# Flexible Ceramic Film Sensors for Free-Form Devices

**DOI:** 10.3390/s22051996

**Published:** 2022-03-03

**Authors:** Tomohiko Nakajima, Yuki Fujio, Tohru Sugahara, Tetsuo Tsuchiya

**Affiliations:** 1 Advanced Manufacturing Research Institute, National Institute of Advanced Industrial Science and Technology, Ibaraki 305-8565, Japan; tetsuo-tsuchiya@aist.go.jp; 2 Sensing System Research Center, National Institute of Advanced Industrial Science and Technology, Saga 841-0052, Japan; yuki-fujio@aist.go.jp; 3 Department of Energy and Environmental Materials, SANKEN, Osaka University, Osaka 567-0047, Japan; sugahara@sanken.osaka-u.ac.jp

**Keywords:** flexible ceramic sensors, free-form devices, 3D electronics, photocrystallization, low-temperature ceramic film growth, wearable devices

## Abstract

Recent technological innovations, such as material printing techniques and surface functionalization, have significantly accelerated the development of new free-form sensors for next-generation flexible, wearable, and three-dimensional electronic devices. Ceramic film sensors, in particular, are in high demand for the production of reliable flexible devices. Various ceramic films can now be formed on plastic substrates through the development of low temperature fabrication processes for ceramic films, such as photocrystallization and transferring methods. Among flexible sensors, strain sensors for precise motion detection and photodetectors for biomonitoring have seen the most research development, but other fundamental sensors for temperature and humidity have also begun to grow. Recently, flexible gas and electrochemical sensors have attracted a lot of attention from a new real-time monitoring application that uses human breath and perspiration to accurately diagnose presymptomatic states. The development of a low-temperature fabrication process of ceramic film sensors and related components will complete the chemically stable and reliable free-form sensing devices by satisfying the demands that can only be addressed by flexible metal and organic components.

## 1. Introduction

**Figure 1:**
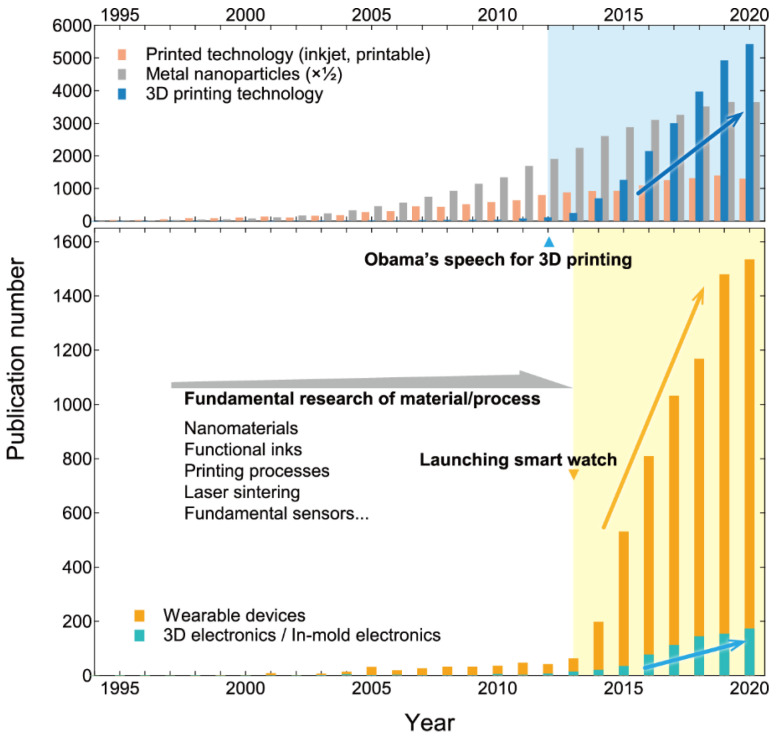
A change in publication numbers of the recent research topics “wearable devices” and “3D electronics/In-mold electronics”, and their related research areas “printed technology, metal nanoparticles, and 3D printing technology”.

A material’s flexibility has recently gained significance in the development of next-generation electronic devices [1]. Numerous electronic devices that have enriched our lives are constructed by the complex assembly of electronic components. The numerous components are installed on metal circuits fabricated on plate-like substrates and integrated into designed housings. For the past half-decade, the manufacturing concept has been fundamentally transformed. Recent advances in the manufacturing process have enabled devices to be designed with a high degree of form freedom. Figure 1 shows a change in the number of publications for the recent research topics “Wearable devices and three-dimensional (3D) electronics/In-mold electronics” and their related research areas. Wearable devices as sensor-integrated systems are primarily used in healthcare management to enhance a healthy life expectancy and are also highlighted by real-time patient monitoring for medical care [2,3,4,5,6,7]. Since 2000, there has been an increase in the number of publications on this topic, which has increased further since 2014. This is related to the release of wearable smart devices capable of real-time vital monitoring with user-friendly management software, such as the first-generation “Apple watch” (Apple Inc., Cupertino, CA, USA) in 2013 and the “Fitbit Force” (Fitbit Inc., San Francisco, CA, USA) in 2014.

This industrial accomplishment provides additional impetus to study materials and fabrication processes in order to develop wearable devices to a higher level of comfort and accuracy in sensing [8,9,10]. The majority of current wearable devices are constructed by assembling ceramic, chip-type electronic components on plate-like substrates [11,12,13]. They are worn directly on our bodies in order to sense various vital signals. As a result, it must be designed with comfort in mind, and one of the primary topics to achieve comfortable mounting on the body surface is device flexibility. Patch-type, skin-mountable devices, for example, have been extensively studied [10,14,15,16,17]. This demands new fundamental research to form functional thin films directly on plastic, flexible, “free-form” substrates. Printing nanoparticles onto plastic substrates has a high affinity for the aim of film fabrication onto plastic substrates, which is known as printed electronics [18,19,20]. Printable electronics primarily require two technology components: (1) nanomaterials as printing materials [21]; and (2) fine-pitch printing methods such as inkjet printers [22,23,24]. Both fundamental studies have already been developed since approximately the year 2000. Therefore, current research for next-generation wearable devices can be driven by markets and the accumulation of associated basic knowledge.

Another important research topic to discuss is the recent advancement of “free-form” devices. 3D electronic devices are one of the most attractive research topics in materials engineering [25,26,27,28,29,30]. These 3D modeling technologies have primarily been advanced on the basis of two-dimensional inkjet printing and the related additive manufacturing methods [31,32,33,34,35,36], coming to fruition as 3D printers. 3D printers first revolutionized resin and metal modeling, and have recently advanced to ceramics and biomaterials. In addition to 3D printing, in-mold methods for producing affordable 3D forms are becoming highly attractive [37,38,39]. These new novel methods for fascinating 3D forms can be very exciting platforms for surface modification to achieve the real integration of shape and multi-functions, and they have been actively studied since 2012. On these 3D electronics/in-mold electronic devices, various sensor components are used, which are currently installed as ceramic chip forms [25]. Direct formation of sensor thin films on the 3D forms, on the other hand, is expected to maximize the functions of the highly designed shapes [40,41].

**Figure 2:**
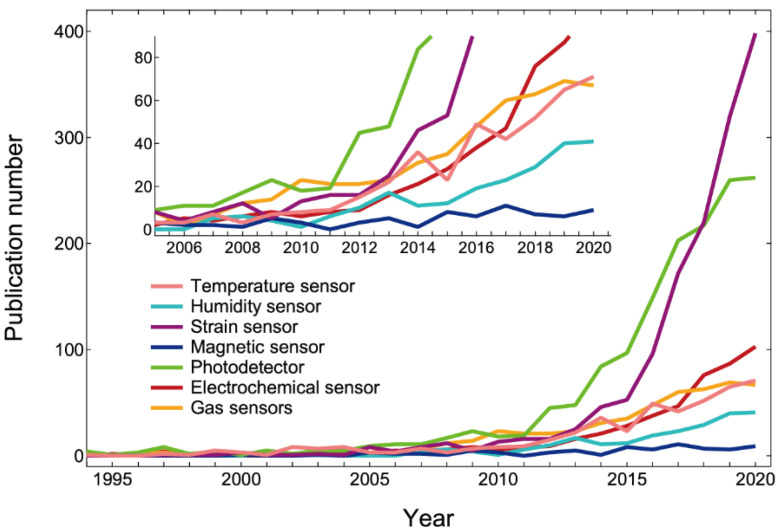
A change in the publication numbers of various types of flexible sensors.

Flexible sensor research is currently being heavily pushed as a result of technological innovations and significant industry demands. Figure 2 shows a change in the number of publications for flexible sensors, and most components have been widely studied since around 2014. This demonstrates a clear correlation with wearable and 3D electronic device research trends. Among the flexible sensors, strain sensors for precise motion detection, such as that of human muscles [42], and photodetectors for biomonitoring, such as continuous glucose monitoring [43,44,45], have seen the most research growth, while other fundamental sensors for temperature [46,47,48,49,50,51,52,53] and humidity [54] have also begun to grow. Temperature sensors, in particular, are the most important since they function as temperature compensators for the other sensors. Recently, flexible gas [55,56,57,58] and electrochemical [59,60,61] sensors have attracted a lot of attention from a new, real-time monitoring application that uses human breath and sweat to accurately diagnose presymptomatic states. Whereas research on flexible magnetic sensors is still in its early stages [62], the significance of entirely new applications such as biomagnetism, derived from the muscles and the brain, will develop.

Through the recent extensive research for flexible sensors, many organic components have been used in place of ceramic sensors since the ceramic components have been difficult to fabricate directly onto flexible plastic substrates due to the high process temperature. However, ceramic components now remind us of the importance of their high property and durability. Therefore, flexible, thin ceramic components have emerged as one of the most important topics in the rapidly expanding field of flexible sensor research, with the goal of realizing chemically stable sensors that are reliable in the long term. The research of functional ceramic film components is now widely expanding target materials to non-oxides like chalcogenides, whereas they have mainly indicated oxide materials so far. The development of fabrication of flexible, ceramic sensor films on plastic substrates would be directly linked to the fabrication of not only sheet-type flexible devices, but also 3D-shaped devices. To realize “free-form” ceramic film sensors and their related components, which are fabricated on shape-agnostic substrates, maximizes the potentials of elaborately designed, free-form device functions. In this article, we will focus on flexible ceramic films fabricated on plastic substrates and outline the current advancements in flexible ceramic film sensors, their related components, and the fabrication process.

## 2. Sensor Materials and Properties

### 2.1. Thermistor Temperature Sensors

**Figure 3:**
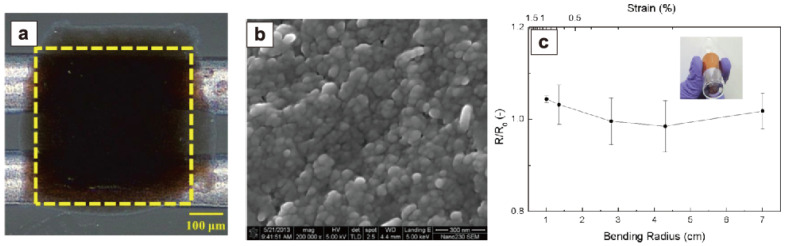
(**a**) Printed NiO film over silver tracks on a glass plate at 25 °C. (**b**) The SEM image of the NiO thin film. (**c**) Electrical resistance variation with the bending radius for printed thermistors on a polyimide film at 50 °C. Reprinted with permission from Ref. [52]. Copyright 2013 American Chemical Society.

Temperature is the most fundamental and essential monitoring value. It is necessary for healthcare, with skin-mountable sensing devices accurately diagnosing presymptomatic states by the real-time monitoring of body temperature and other vital indicators, such as blood glucose levels and cardiac rate. Temperature calibration using temperature sensors can properly measure these sensing target values, demonstrating the significance of temperature sensing. Thermistors are one of the most popular temperature sensors, as they monitor the temperature variation of resistance. Huge numbers of ceramic chip thermistors have been applied in electronic devices, and the sensitivity is evaluated by the thermistor constant *B* ($R=R_{0}\;exp(B/T)$), where *R*_0_ is the resistance at an infinite temperature and *T* is the temperature). Table 1 lists the *B* constant, substrate, and fabrication process for flexible thermistors that have recently been reported. Although conventional thermistors have always been used in ceramic chip forms, researchers have recently attempted to produce flexible ones on plastic substrates by overcoming their high process temperature (>800 °C). Huang et al. used an inkjet printing technique to fabricate NiO flexible thermistors on polyimide (PI) sheets with a high thermistor constant (*B*) at 4300 K [52]. As shown in Figure 3, they were able to create compact and bendable thermistor films by using the low crystallization temperature binary oxide NiO, and very uniformly small-sized (ca. 50 nm) nanoparticle dispersion. These NiO nanoparticles that were printed via inkjet were calcined at a very low temperature of 200 °C. The heated NiO nanoparticles showed good electrical connection as a thermistor film, and they confirmed that the resistance of bent thermistors maintained nearly the same value as that of flat thermistors with a variation of less than 10% within the bending radius of 1.0–7.0 cm.

**Figure 4:**
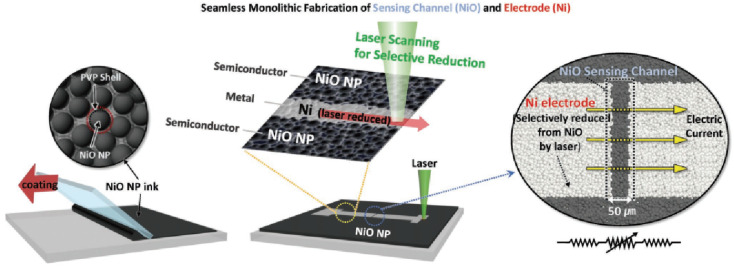
Schematic of the monolithic laser reductive sintering process. NiO nanoparticle ink is coated on a substrate by the doctor blading technique. The selective laser irradiation of the dried NiO layer using a computer-aided galvano-mirror system and the monolithic Ni–NiO–Ni structure, having several tens of micrometer-wide NiO-channels, was formed by the simple hatching technique. Reprinted with permission from Ref. [46]. Copyright 2020 Wiley-VCH.

Shin et al. demonstrated a new method for fabricating flexible oxide thermistors by combining techniques of nanoparticle dispersion coating and monolithic laser-induced reductive sintering. Doctor Blading was used to coat the NiO nanoparticles dispersion onto polyethylene terephthalate (PET) substrates, and the coated precursor films were then dried under ambient conditions. The dried NiO coating was reduced, in part, by a 532 nm continuous wave laser [46]. The irradiated part was converted to Ni metal, and metal electrode patterning was achieved using laser scanning (Figure 4). The obtained flexible NiO thermistors showed a very high *B* constant at 8162 K, and they demonstrated body skin temperature monitoring by using fabricated epidermal NiO thermistor sensors for healthcare applications.

Conventional thermistor materials, such as Mn-spinel and Mn, Co-perovskite oxides, have a very high process temperature above 800 °C [66]. As a result, NiO has been used to form films on plastic substrates at a very low process temperature. Fujita et al. developed a new material that can be adapted to a low process temperature in order to investigate new flexible thermistors. They used RF magnetron reactive sputtering to prepare wurtzite Al_0.85_Ti_0.15_N*_y_* thin films on polyimide substrates, and the obtained thermistor films showed a *B* constant at 2525 K [51]. The halide perovskite is, likewise, a very new material system. Li et al. presented inkjet-printed Cs_2_SnI_6_, a new thermistor material with a *B* constant at 4400 K. This material may be formed solely through the printing and drying (at 120 °C) processes. Based on this characteristic, they developed fabric-based thermistors for wearable devices, which may be realized by infiltrating a starting solution into the polyester fabric [64].

**Figure 5:**
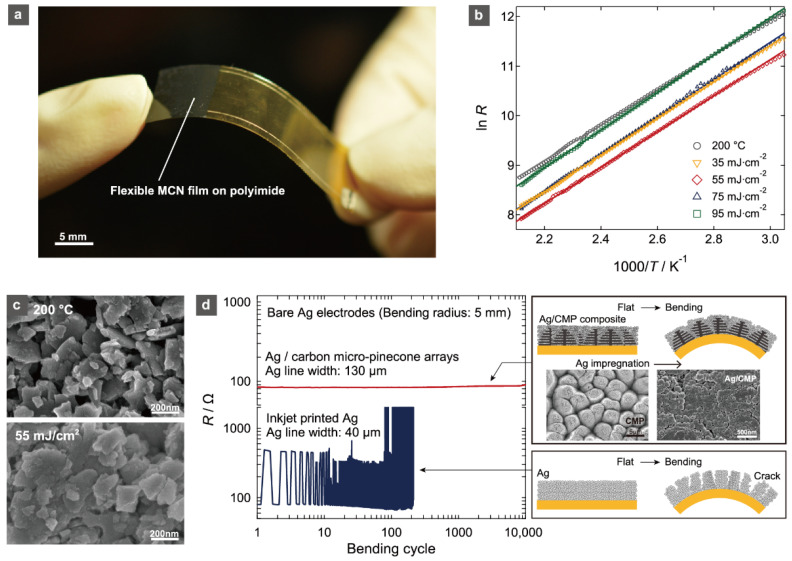
(**a**) Photograph of the MCN film thermistor that was prepared at 55 mJ·cm^2^ of Kr laser irradiation on the PI sheet with Ag/carbon micro-pinecone (CMP) composite electrodes. (**b**) Temperature dependence of *R* (Ω) at 27–200°C for the MCN films on PI substrates prepared by drying at 200 °C and laser irradiation at 35–95 mJ·cm^−2^ for 600 pulses. (**c**) FESEM images of the surface morphology of MCN films prepared by drying at 200 °C, and by KrF laser irradiation at 55 mJ·cm^−2^ for 600 pulses. (**d**) Changes in resistance (*R*) during bending with a bending radius of 5 mm for patterned lines of the Ag/CMP composite and inkjet-printed Ag. Schematics of the Ag electrodes with, and without, CMP arrays on the flexible substrates in flat and bent states with FESEM images of patterned CMP and Ag/CMP composite on the polyimide substrate. Reprinted with permission from Ref. [49]. Copyright 2017 American Institute of Physics.

Nevertheless, the use of conventional reliable materials with high crystallization temperatures, such as Co- and Ni-codoped spinel Mn_3_O_4_, has been strongly demanded by the industry due to their proven high thermistor properties and chemical stability. Nakajima et al. used a photocrystallization technique to solve the high process temperature issue [48,49,65,67]. Figure 5 shows the crystallization of the spinel oxide thermistor Mn_1.56_Co_0.96_Ni_0.48_O_4_ (MCN) by KrF laser irradiation to MCN-nanoparticle-deposited films on polyimide substrates. The resistivity of laser-irradiated MCN films decreases owing to the crystallite growth and intergrain connections (Figure 5b,c). The *B* = 3633 K of the developed MCN flexible thermistor films was comparable to that of commercial MCN-based chip thermistors. The flexibility of thermistors has been greatly improved by the use of very stable bottom electrodes against bending motion. KrF laser irradiation was used to create carbon micro-pinecone (CMP) arrays [68], and the laminated micro-carbon nanosheets in the CMP were impregnated with Ag nanoparticles. By establishing electric connections, the composite Ag/CMP was significantly stabilized for bending (10,000 bending cycles at a 5 mm bending radius) (Figure 5d). The stabilizing bottom electrodes resulted in the reliable operation of flexible MCN thermistors [49].

**Figure 6:**
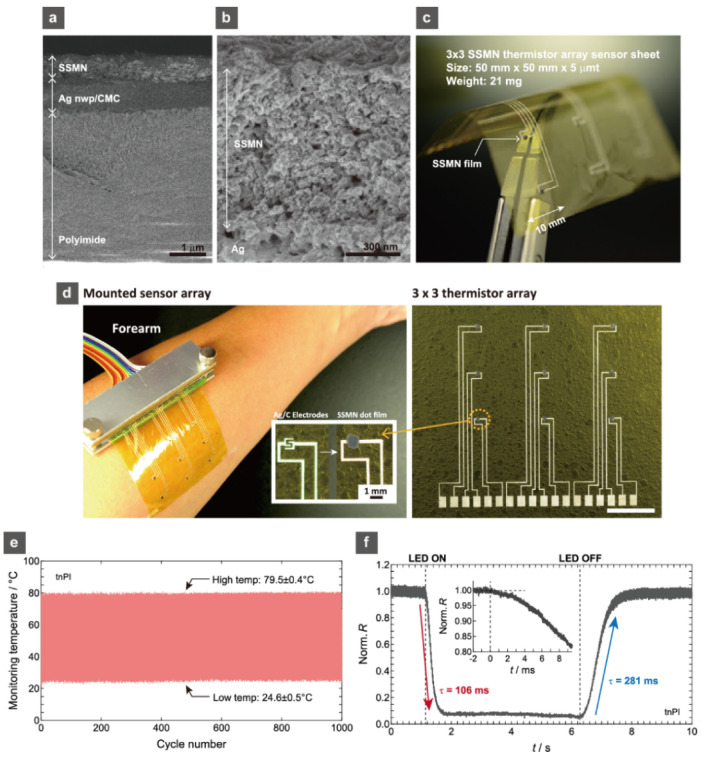
(**a**) FESEM image of the SSMN thermistor film on the thin PI substrate with the Ag nwp/CMC bottom electrode layer. (**b**) Enlarged FESEM image of the SSMN layer on the thin PI substrate. (**c**) Photographs of the 3 × 3 array patterns of the SSMN thermistor sub-millimeter sized (900 μm diameter) films on the 5 × 5 cm thin PI sheets with the Ag nwp/CMC bottom electrode patterns and (**d**) the mounted sensor array film on the human forearm. (**e**) Temperature cycling test for the SSMN thermistor film on thin PI for 0–1000 cycles. (**f**) Normalized *R* variation of the SSMN thermistor film on thin PI during UV LED illumination. Reprinted with permission from Ref. [53]. Copyright 2020 American Chemical Society.

Based on the photocrystallization technique, the formation of perovskite oxide thermistors on PI substrates has also been realized [53]. The PI substrates are only 5 μm thick, and the KrF laser irradiation crystallized the printed perovskite oxide Sr-and Ni-doped SmMnO_3_ (SSMN) precursor films (Figure 6a,b). Composites of laser-carbonized microcone (CMC) arrays and Ag nanowires/nanoparticles (nwp) were used to form the bottom electrodes. The obtained perovskite thermistor array sheet was only 21 mg in weight and bendable to 180°. The ultrathin thermistor array sheet allowed for comfortable human skin attachment (Figure 6c,d). The temperature cycle test (between 24.5–79.5 °C) for 1000 cycles demonstrated thermistor sheet stability, and the thinness (5 μm) achieved a very high-speed response at *τ* = 106–281 ms owing to the small heat capacity. By sufficiently decreasing the process temperature for oxide ceramics, the photocrystallization process was able to broaden the material options.

### 2.2. Humidity Sensors

Humidity, like temperature, is a fundamental sensing property. Respiratory monitoring is an important measurement in biomonitoring in medical diagnoses. There are two primary methods for measuring humidity: (1) resistive humidity sensing; and (2) capacitive humidity sensing. In each case, surface-adsorbed water molecules vary the resistance or capacitance of semiconductor materials. Whereas capacitive polymer materials have been extensively used in humidity sensors, the response speed and long-term stability are the main issues that need to be addressed in order to improve the real-time monitoring. Carbon-based materials, such as graphene oxide and nanostructured carbon, have thus far outpaced inorganic materials in research [68,69,70]. Some inorganic nanomaterials, such as WO_3−*x*_, black phosphorous, and WS_2_ nanosheets, have recently been demonstrated to be good humidity sensors [71,72,73]. Table 2 lists their humidity sensing parameters. Among inorganic humidity sensors, low-dimensional nanomaterials are particularly advantageous for sensitivity and response speed.

**Figure 7:**
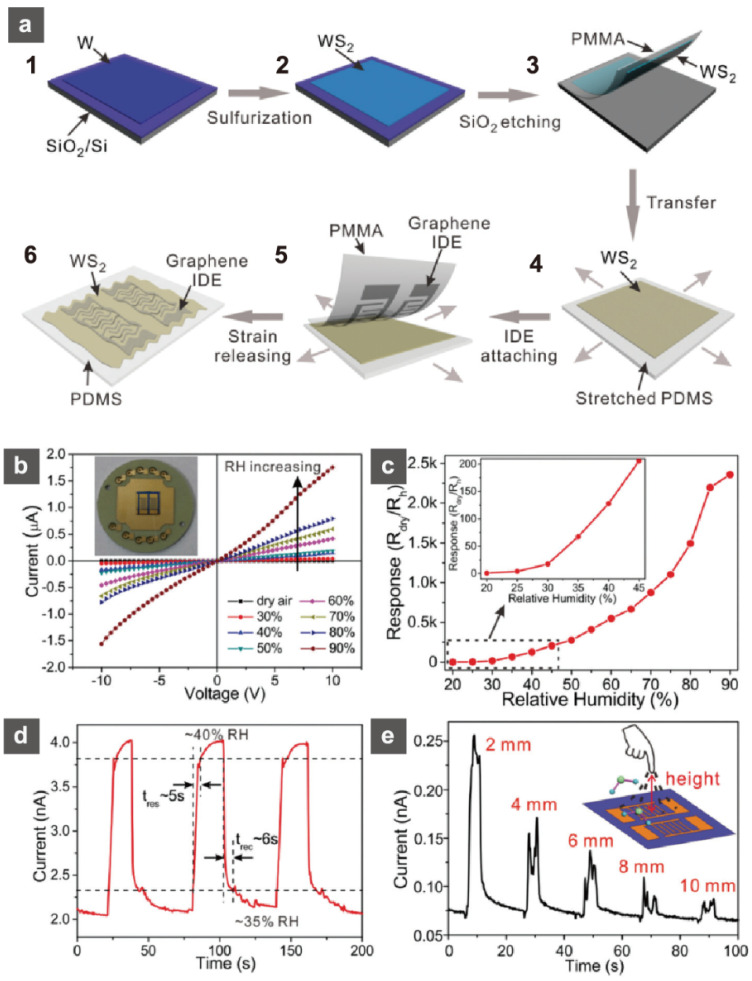
(**a**) Schematic of the device fabrication process. Humidity sensing properties of the WS_2_ film on the SiO_2_/Si substrate. (**b**) Current–voltage (I–V) characteristics in different relative humidities. Inset: Image of a rigid WS_2_ sensor connected to the sample stage by wire bonding. (**c**) Response of the sensor at different relative humidities (RHs). (**d**) Time-dependent response current of three cycles of humidity switching between the low RH level (35%) and high RH level (40%). (**e**) Time-dependent response current with a fingertip vertically approaching and retracting away from the device surface at different distances. The RH of the test environment is about 40%. The RH near the finger is about 43%. Reprinted with permission from Ref. [72]. Copyright 2017 Royal Society of Chemistry.

At 2357% (20–90% relative humidity (RH)), atomically thin, two-dimensional (2D) layered semiconductor WS_2_ films showed very high sensitivity. Guo et al. suggests that the sulfurized, polycrystalline few-layer WS_2_ film has higher sensitivity than that of a highly crystalline, monolayer WS_2_ film, indicating a grain-boundary-assisted gas sensing mechanism [72]. The response speed was remarkably fast. Response and recovery times were 5 s and 6 s, respectively. This fast response is a key feature of 2D-layered semiconductors. Sulfurization of a tungsten film on a SiO_2_/Si substrate and transfer to a stretchable polydimethylsiloxane (PDMS) substrate produced the WS_2_ film (Figure 7a). Figure 7b–d demonstrate that the conductivity of WS_2_ increased sensitively over a large RH range (~90%). The time-dependent response of current while a fingertip was vertically approaching and retracting away from the device surface at different distances revealed a definite response to moisture from the fingertip. The resulting WS_2_ sheet was used as a transparent, flexible, and stretchy humidity sensor by using graphene as electrodes on a thin PDMS substrate. Mounting this stretchable humidity sensor onto the skin behind the nose further proved the detection of aspiration. Thus, recent achievements in low-dimensional nanomaterials have aided in the development of flexible humidity sensors, and their practical realization will follow soon after additional improvements in sensor reliability, such as mechanical and chemical stability, as well as bending resistance.

### 2.3. Strain Sensors

Flexible strain sensors can be used in a variety of sensing applications that take advantage of their flexibility and large-area manufacturing, such as mechanical deformation of the human body [17,80], changes in external natural forces [81,82], and health monitoring for social infrastructures [83,84,85]. Takei et al. reported electrical muscle stimulation (EMS) using textile electrodes, and mechanomyogram (MMG) sensor measurement using an ultrathin piezoresistive silicon strain sensor in flexible form, among other applications of strain sensors for mechanical deformation of the human body [80]. This sensor is made entirely of flexible materials, including 5 μm of thick piezoresistive silicon, stretchable conductive paste, a polyimide substrate, and silicone rubber. Because of the thin layer and flexible components, the Si chip did not break, even when the sensor was bent. This indicates that the sensor can be fixed and used along the curved surface of the human body. The sensor confirmed that muscle contraction differs depending on the magnitude and frequency of the EMS voltage.

Xu et al. have developed a thermal sensor for monitoring wind speed and direction in the context of a sensing application for external natural forces [81]. Kanazawa et al. subsequently showed that a novel flexible sensor based on a strain sensor matrix can detect wind pressure distribution [82]. The strain sensor matrix was printed on the film’s surface to detect the deflection of the suspended structures via whole-screen printing. This sensor was designed to have individual mechanical displacements of the shaped film that was processed to be mechanically movable. Using the original software, the obtained wind pressure distribution of a cold wind stream at a wind speed of 4.0–8.0 m/s from a hairdryer to the sensor sheet was graphically imaged. Since the control of air flow and air resistance against curved surfaces is expected to contribute to the development of various industrial domains, including automobiles and aircraft, monitoring wind pressure distribution is expected to contribute.

**Figure 8:**
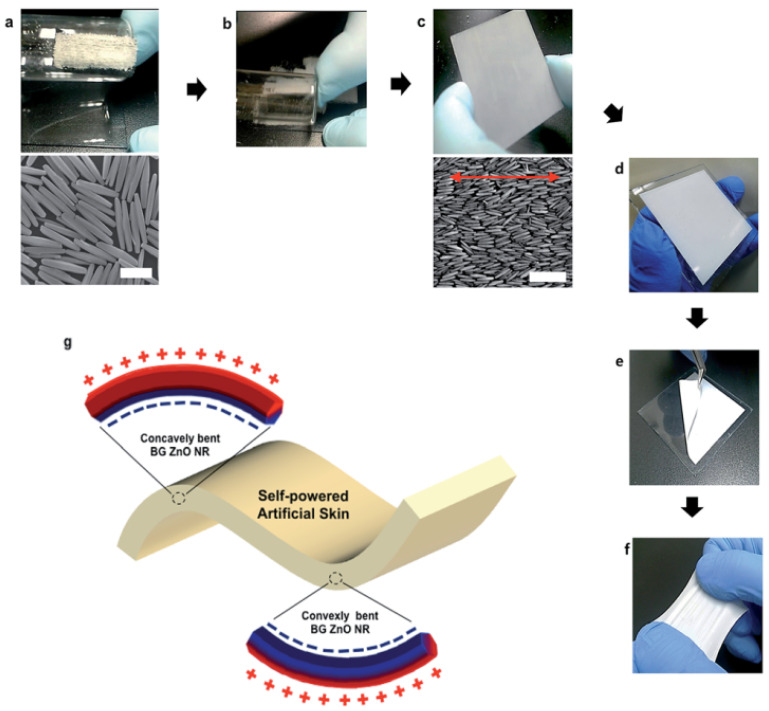
Self-powered artificial skin (SPAS) fabrication with a bi-axially grown ZnO nanorod array on a PDMS film through one-direction rubbing. (**a**) Bi-axially-grown ZnO NR powder on a velvet cloth, which was attached to one side of a round glass tube. The bottom image shows bi-axially-grown (BG) ZnO NRs from a hydrothermal synthesis after thermal annealing at 400 °C for 2 h (scale bar = 2 μm). (**b**) BG ZnO NRs rubbing in one-direction on a PDMS coated slide glass. (**c**) Single monolayer formation of BG ZnO NRs through rubbing. The bottom image indicates a nematic-like array of the BG ZnO NRs (red arrow indicates the rubbing direction; scale bar is 6 μm). (**d**) Seven layers of the SPAS on a glass substrate. (**e**) Detachment of the SPAS from the glass substrate. (**f**) Photograph demonstrating how the SPAS is flexible (in (**e**)) and stretchable. (**g**) Schematic 3D diagram describing the piezoelectric potential generation of a bent SPAS. Reprinted with permission from Ref. [17]. Copyright 2014 Royal Society of Chemistry.

The range of strain sensor application domains has also stimulated research into ceramics-based strain sensors. Lee et al. have proposed a self-powered artificial skin (SPAS) without electrical wiring as one feasible solution for electrical power in wearable strain sensors for human motion (Figure 8) [17]. This is built on a piezoelectric nano generator with high mechanical stretchability, narrow thicknesses, environmental compatibility, and a large area. The SPAS, which has a large area of bi-axially-grown (BG) zinc oxide (ZnO) nanorods (NRs), fabricated via a rapid dry rubbing process, can supply the electrical energy for implanted biomedical devices via mechanical bending motion. The SPAS is fabricated to be piezoelectrically activated along the polarity from the deformation. The bending mode of BG ZnO NRs embedded in PDMS is varied convexly/concavely, and a bent BG ZnO NR receives transverse shear stress–stain. Both positive and negative piezoelectrical potentials are generated over the transversal direction.

**Figure 9:**
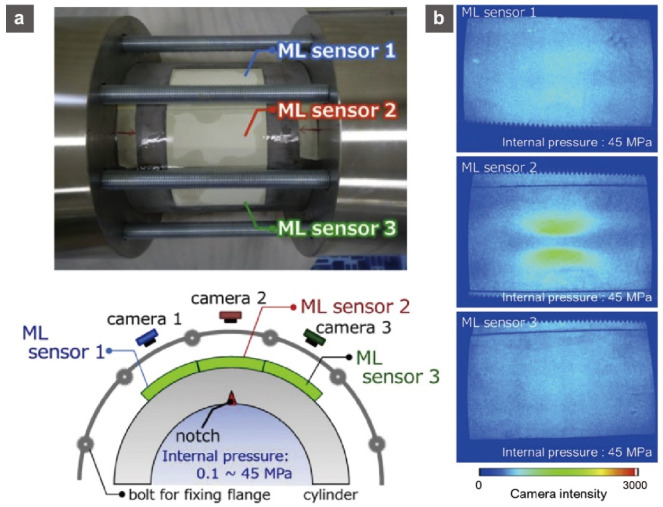
(**a**) Photograph of a storage cylinder with three attached mechanoluminescence sensors and a schematic view of the mechanoluminescence image monitoring system. (**b**) Mechanoluminescence images obtained from each of the mechanoluminescence sensors 1–3 attached to the outer surface of the storage cylinder at the internal pressure of around 45 MPa in the 51st cycle of fatigue testing. Reprinted with permission from Ref. [83]. Copyright 2016 Elsevier.

Global attention is being drawn to health monitoring for social infrastructure in order to ensure effective maintenance and avoid the recurrence of severe disasters. We recognize that there is a high demand for efficient techniques for dynamic and precise stress/strain imaging on large-scale structures. To address this difficult challenge, new types of sensors based on flexible sensors with conductive nanostructures, photoelectric methods of assembling nanowires or nanotubes, or microstructured rubber layers were developed. Flexible mechanoluminescent (ML) (or elasticoluminescent, piezoluminescent) sheets using ML materials and optical resin stand out among different new types of sensors for monitoring stress and strain distribution on large-scale structures [83,84,85]. Even in the elastic deformation region, the ML material emits intense light on a regular basis, accompanied by mechanical operations such as deformation, friction, and impact, with the light intensity being proportional to strain energy. Thus, by recording the ML distribution on the ML sheet attached to the object surface, the stress/strain distribution of the object may be observed. For example, Fujio et al. reported that a mechanoluminescent SrAl_2_O_4_:Eu-based ML sheet sensor detected cracks emanating from the inner cracks of a high-pressure hydrogen storage cylinder in a hydraulic pressure cycling test with a maximum pressure of 45 MPa (Figure 9) [83]. Furthermore, based on the ML pattern analysis and mechanical simulation using the finite element distribution, the distance between two points with high equivalent strains on the pressure vessel’s surface was found to be inversely related to the crack depth. This indicates that the ML distribution pattern can propose a non-destructively quantified visualization technique for the growth behavior of the inner crack (the residual life of the pressure vessel) [85].

### 2.4. Gas Sensors

New biomonitoring applications, such as aspirated air and skin gas sensing for presymptomatic diagnosis of the human body, are attracting a large number of wearable flexible gas sensors [86,87]. So far, oxide-based gas sensors have been extensively studied. Due to developments in nanomaterials, the sensitivity of various gases, such as acetone, formaldehyde, and H_2_S, has increased [88,89,90,91,92], and many researchers are focusing on room temperature operation and device fabrication using nanostructured sensor films [57]. Recently, state-of-the-art nanostructured flexible gas sensor films based on nanoceramic were reported. Table 3 shows the gas-sensing properties and morphology of nanoceramic flexible gas sensors.

**Figure 10:**
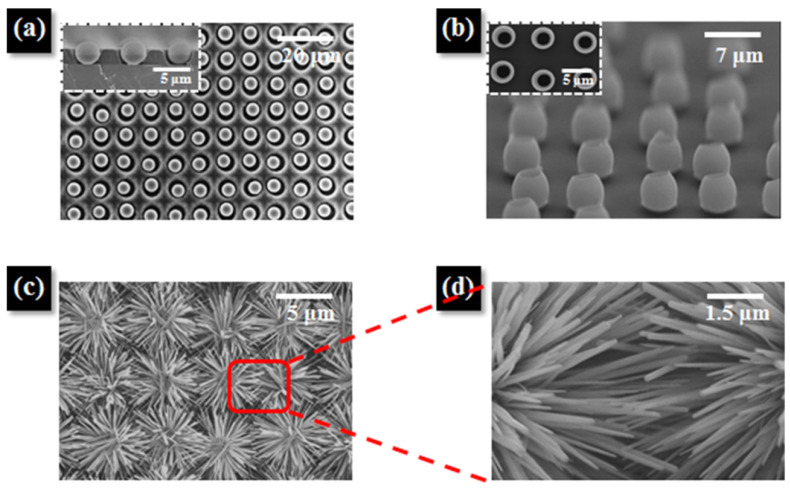
(**a**) Top-view SEM image of a microparticle array in circular well patterns. Inset: Cross-sectional SEM image showing microparticles arrayed in height-optimized well patterns. (**b**) Tilted-view SEM image of aligned ZnO shells after transfer and calcination at 250 °C for 3 h in air on a hot plate. Inset: Top-view SEM image showing polystyrene-removed ZnO shells after calcination. (**c**) SEM image of the ZnO nanoflower network structure after growth of ZnO NRs in- and outside the shells, and (**d**) magnified SEM image of the junctions between the NRs. Reprinted with permission from Ref. [96]. Copyright 2017 American Chemical Society.

Among the ceramic-based gas sensors, ZnO nanomaterials have already been extensively studied for various gas species such as H_2_, NO_2_, NH_3_, O_2_, and CO by the large resistive response against those gas adsorptions. Figure 10 shows micropatterned double-faced ZnO nanoflowers fabricated on PI substrates for flexible NO_2_ sensors. Kim et al. created well-aligned ZnO nanoflowers by first forming a microparticle array, then sputtering ZnO, transferring it to a PI substrate, and, finally, growing ZnO nanoflowers in aqueous solution [96]. The fabricated sensor devices demonstrated high selectivity for NO_2_ at 270 °C, with a stable response value (resistance ratio after and before exposure to the target gas) of 218.1. The sensor’s response and recovery times were 25.0 s and 14.1 s, respectively. These good sensing properties result from a combination of high surface area, numerous active junction points, and donor point defects in the ZnO nanoflowers. They also exhibited the ZnO nanoflower gas sensors’ exceptional mechanical stability. Despite some physical degradation of the device following the bending test, the sensor with a 3D micropatterned network structure continued to function even after 10,000 cycles with a curvature radius of 5 mm. The results of these types of highly ordered micro- and nanostructures are very important for demonstrating the advantages of increasing surface area for flexible gas sensors while maintaining bending resistance.

**Figure 11:**
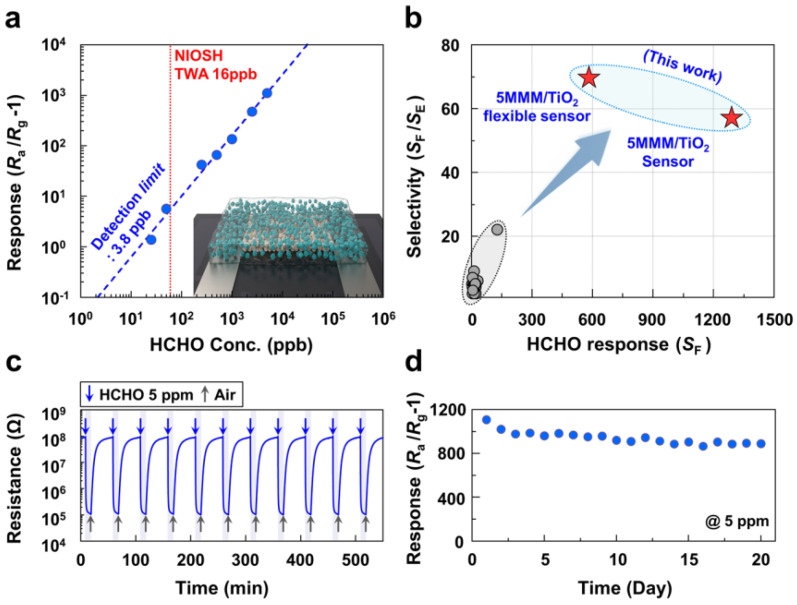
(**a**) Gas response as function of formaldehyde concentration. (**b**) Formaldehyde selectivity and response compared to the reported values in the literature. (**c**) Repeated sensing transients to 5 ppm of formaldehyde at 23 °C under 365 nm UV radiation. (**d**) Long-term stability of the MMM/TiO_2_ sensor (UV light illumination during sensor measurement). Reprinted with permission from Ref. [110]. Copyright 2021 Springer Nature.

Regardless of sensor adaptability, room temperature operation has recently been one of the hottest topics in gas sensors. However, it has particular significance in flexible gas sensors for skin-mounting devices. As shown in Figure 11, Jo et al. investigated the ultrasensitive detection of formaldehyde at room temperature using flexible TiO_2_-based chemiresistive sensors [110]. The sensor was fabricated using the following procedures: To fabricate the pristine TiO_2_ sensor (thickness: 2 μm), the TiO_2_ slurry was screen-printed on a PET substrate with two Pt/Ti interdigited electrodes and then heat treated. Spin coating on the TiO_2_ film resulted in the formation of a mixed matrix membrane (MMM) (zeolitic imidazole framework (ZIF-7)/polyether block amide (PEBA) composite material) (thickness: 200 nm). The obtained MMM/TiO_2_ sensor demonstrates a highly selective detection of formaldehyde and ethanol under UV illumination (wavelength: 365 nm, radiation intensity: 225 mW) with negligible cross-responses to other indoor pollutants. By molecular sieving, the coating of MMM consisting of ZIF-7 nanoparticles and polymers on TiO_2_ sensing films removed ethanol interference, enabling an ultrahigh selectivity (response ratio > 50) and response (resistance ratio > 1100) to 5 ppm of formaldehyde at room temperature. They also put the sensors’ strengths through mechanical testing. The response characteristics to formaldehyde were investigated using a flat sensor configuration at bending angles ranging from −155 to 155°. The sensing response was almost completely unaffected by the bending angles. Moreover, even after 200 bending cycles, the sensing transient behavior for 5 ppm formaldehyde remained unchanged. They stated that the robustness of the sensor’s resistance to bending motion can be attributed to its unique design, which includes a TiO_2_ detecting layer sandwiched between two polymeric layers (PET and MMM). The synergistic combination of the highly permeable and uniform molecular-sieving overlayer using a unique metal organic framework (MOF)-polymer composite membrane and UV light-enhanced selective gas sensing reaction at room temperature resulted in the formaldehyde sensing behavior in this study. The improvement of gas sensor properties at room temperature will significantly improve real-time monitoring of human health using flexible sensing devices.

**Figure 12:**
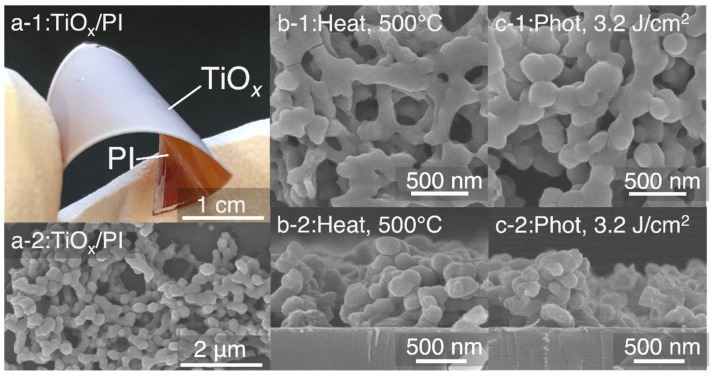
(**a**) Photograph and top view of the SEM images of TiO*_x_* nanostructure thin film on PI substrate after photosintering at 3.2 J·cm^−2^ once on the PI substrate. Top view/cross-sectional view of the SEM images of TiO_2_ nanostructures on the rigid SiO_2_ glass substrate after (**b**) heat sintering at 500 °C and (**c**) photosintering at 3.2 J·cm^−2^ one time. Reprinted with permission from Ref. [58]. Copyright 2020 American Chemical Society.

Sugahara et al., tried to fabricate flexible gas sensors for volatile organic compounds sensing damage-less processes on plastic substrates using a room temperature photosintering method with high-intensity pulsed light (HIPL). The TiO_2_ nanostructures were directly produced on a plastic substrate using the metal-organic deposition method, HIPL irradiation, and sintering. Figure 12a shows the photograph, as well as a top view of SEM images of the direct growth of the TiO_2_ nanostructure thin film on a polyimide (PI) substrate using titanium oxide as a precursor [58]. With the HIPL systems, the photoirradiation energy of 3.2 J/cm^2^ (300 μs) is used only once. Figure 12b,c show the top and cross-sectional views of the SEM images of TiO*_x_* nanostructures on the SiO_2_ silica glass substrate after 500 °C for 15 min and photosintering at 3.2 J/cm^2^, respectively, for heat sintering. Both photosintering with HIPL at the boundaries and thermal heat sintering are used to diffuse and neck the interface between the particles. The obtained flexible gas sensors demonstrated an extraordinarily quick sensing response and recovery time due to a large surface area to volume ratio and a highly crystalline nanostructure. With increasing formula weight of the gas species, the average sensor sensitivity increased in the order of methanol < ethanol < 1-propanol vapor, which may involve the redactive nature of the gas species due to its dependence upon the number of CH chains.

### 2.5. Electrochemical Sensors

Flexible electrochemical sensors, like gas sensors, are gaining traction in healthcare monitoring. Glucose sensors have generated considerable interest as real-time monitoring and prophylaxis devices for diabetics using flexible, patch-type devices. Flexible electrochemical glucose sensors based on nanoceramics thin films and oxide/carbon nanocomposite films have been reported [111,112,113,114,115,116]. Archana et al. reported the development of flexible nonenzymatic electrochemical glucose sensors based on an orchestrated network of Cu/Ni MOFs in an octahedron shape [112]. CuO/NiO spherical nanoparticles are uniformly disseminated and tightly pinned with hierarchical carbon using a Cu/Ni MOF as a template, and the MOFs and metal oxide-carbon nanocomposites are subsequently coated onto plastic tapes to form electrochemical sensor probes for nonenzymatic glucose sensors. The generated Cu(II)/Cu(III) and Ni(II)/Ni(III) redox species facilitate an efficient glucose electrooxidation. Amperometric detection of glucose by the obtained flexible sensors demonstrated a clear response to a wide range of glucose concentrations ranging from 100 nM to 4.5 mM. The sensitivity was 586.7 μA·mM^−1^·cm^−2^, while the detection lower limit was 37 nM. Additionally, the bending resistance was quite considerable. The bending angle at 180° had no effect on the current density in the analysis for 5 mM glucose.

**Figure 13:**
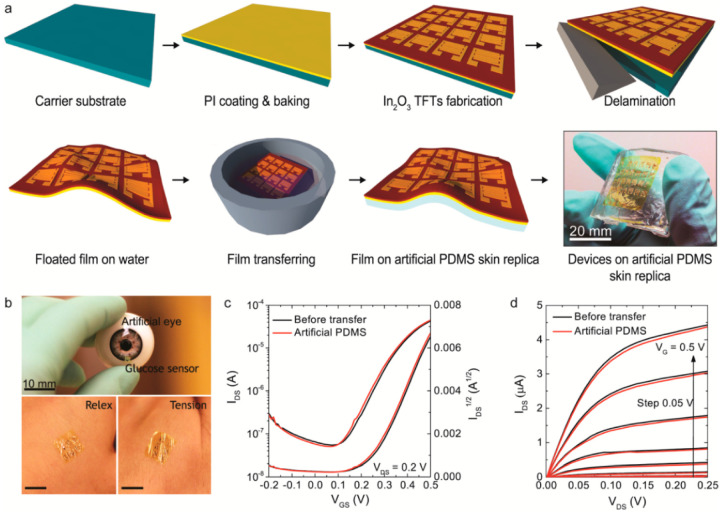
Liquid-gated In_2_O_3_ FET-based conformal biosensors. (**a**) Schematic illustration of the flexible biosensor fabrication procedure. Glass substrates were coated with a thin film of PI. Next, In_2_O_3_ thin films were deposited over the PI via an aqueous solution-phase spin-coating procedure. The In_2_O_3_ thin films were then annealed at 250 °C. Interdigitating Au/Cr electrodes were patterned by photolithography. The PI films with In_2_O_3_ FET arrays were delaminated from the underlying glass substrates and floated in water to unroll the films. A synthetic PDMS skin replica was prepared. Thin-film, In_2_O_3_ FET sensors were transferred to artificial PDMS skin samples. Ultrathin PI films easily contact uneven artificial skin surfaces. (**b**) Conceptual images of conformally contact devices on an artificial eye for glucose sensing in tears are shown. Thin-film sensors remained in contact with the skin even during tension and relaxation. (**c**,**d**) Device performance of thin-film In_2_O_3_ FETs on rigid substrates and flexible artificial PDMS skin substrates is shown. After the transfer of In_2_O_3_ FETs to the skin replica, the devices had similar performance under liquid gating. Low voltage driving and good pinch-off characteristics were observed. Reprinted with permission from Ref. [117]. Copyright 2015 American Chemical Society.

Rim et al. developed a straightforward solution-processing procedure for fabricating ultrathin, highly sensitive In_2_O_3_-based field-effect transistors (FETs) for glucose sensing [117]. Spin-coating the starting solution for In_2_O_3_ resulted in the formation of a nanometer-thick (3.5 nm), smooth, and highly uniform In_2_O_3_ film, which was then transferred to a PDMS skin replica (Figure 13). The FETs based on In_2_O_3_ had mobilities of ~20 cm^2^·V^−1^·s^−1^ and on/off ratios of >10^7^. The fabricated sensor sheet was very well adhered to the rough artificial skin and ocular surfaces. They confirmed that the FET performance was maintained during the transition of the In_2_O_3_-based device to PDMS. The study demonstrates unequivocally that ultrathin oxide films can retain their electrical properties even on very flexible free-form substrates.

### 2.6. Optical and Magnetic Sensors

Furthermore, optical and magnetic sensors will be crucial for next-generation devices, including new optical glucose monitoring and biomagnetic sensing applications. These devices are expected to be used as flexible, patch-type sensors dubbed “*e*-skin devices.” Flexible photodetector sensors have been fabricated using photo-active materials such as ZnO, Ga_2_O_3_, and MoS_2_ [118,119,120,121,122]. Liu et al. demonstrated the fabrication of all-printable flexible polycrystalline ZnO granular nanowires photodetectors [118]. The sensor sheets obtained exhibited a relatively high responsivity (2.6 × 10^7^ at 1 V) and detectivity (3.3 × 10^17^ Jones at 1 V). These high properties originate from band-edge modulation along the axial direction of the granular nanowire. Electrospinning and inkjet printing were used to fabricate the sensors and bottom Ag electrodes, respectively. Basically, flexible photodetectors have been realized through the immobilization of nanoceramics on flexible substrates such as flexible gas sensors. Furthermore, Zheng et al. fabricated flexible ZnO-based photodetectors. They used chemical vapor deposition to prepare ZnO nano-networks, and the resulting ZnO nano-networks formed self-supported, flexible photodetectors [119]. The photodetectors constructed had very fast response rise and decay rates of <0.16 s and <0.12 s, respectively. The photodetectors have primarily been developed using materials with a low crystallization temperature, such as simple binary oxides, such as ZnO and In_2_O_3_, which has facilitated research development in comparison to other flexible sensors since there are multiple options in the fabrication process’s flexibility.

Advanced medical diagnostics, such as heart examinations using magnetocardiography, will lend additional credence to next-generation flexible magnetic sensors. Due to the relatively low signal at roughly 100 pT, magnetocardiography has so far only been performed using a superconducting interference quantum device with a cryogenic apparatus. However, significant development advancements in highly sensitive magnetoresistance (MR) devices based on oxide and alloy-based-thin films have enabled the detection of this low-level magnetic signal using only a DC power source [123]. Flexible sensors have recently been studied in MR and hall resistance devices [62,124,125]. Incorporating these flexible magnetic sensing devices will have a significant impact on next-generation medical diagnosis through the incorporation of wearable devices for real-time monitoring.

### 2.7. Related Components

Since flexible sensor devices include not only sensor elements, but also electrodes, many passive components, integrated circuits, and batteries, research developments into these related components are very important. Research regarding flexible electrodes is more advanced than that of the others. So far, not only flexible metal electrodes [126], but also oxide-based flexible transparent electrodes such as Sn-doped In_2_O_3_ and Ga-doped ZnO, have been extensively studied [127,128,129]. Additionally, research on flexible ceramic resistors (e.g., RuO_2_) [130] and capacitors (e.g., Ba(Zr_0.35_Ti_0.65_)O_3_ and Na_0.5_Bi_0.5_TiO_3_-EuTiO_3_) [131,132,133,134] has been boosted lately by the significant demand from other primary components and new applications. Resistors and capacitors are essential components for any kind of electronic device. Therefore, research progress for the flexibilization of these components, as well as sensor components, is highly demanded. Since many capacitor materials, such as perovskite oxides, have a generally high crystallization temperature, flexible capacitors require the further development of low-temperature production methods. At the moment, they are mostly carried out using a transferring method [135,136], photocrystallization [137], and the use of alternative flexible substrates with high heat resistance, such as mica [132]. The power supply, as one of the most important components for flexible devices, is also being extensively investigated. The following types of flexible power sources are primarily studied: (1) solid state batteries [138,139,140]; (2) solar cells [141,142]; (3) supercapacitors [143]; and (4) piezoelectric nanogenerators [144,145,146]. Many next-generation, free-form devices using flexible ceramic sensor films are now being realized as a result of these strenuous efforts in the development of flexible components.

## 3. Fabrication Process

### 3.1. Challenges and Solutions

One of the major challenges to the practical use of flexible electronics with high functionality and dependability has been the difficulty of fabricating ceramic films on plastic substrates. This is because the process temperature for ceramic films is often relatively high for plastic substrates (>400 °C). As a result, research for fabrication processes has been extensively developed for various kinds of ceramic film materials, and several significant techniques for low-temperature fabrications have been published over the last decade. There are two important processes: (1) photocrystallization; and (2) transferring methods. For the crystallization of precursor films formed on plastic substrates, photocrystallization is carried out using pulsed laser or continuous lamp irradiation. Instead of a conventional furnace’s heating, the energy input is provided by a light source. This process offers the advantages of very simple direct fabrication on flexible substrates and high compatibility with all printed processes. There are numerous transferring methods that have several derivatives. Basically, crystalline films have been prepared on different ceramic substrates. They are transferred using polymer stamping or laser lift-off methods. This method may fabricate high-quality ceramic films, such as epitaxial ones, on flexible substrates, although the process complexity is increased when compared to the general photocrystallization process. Advancements in low-temperature ceramic film production mean that ceramic films can now be used on flexible devices.

### 3.2. Photocrystallization Process

One strong solution has been developed, known as photo-assisted chemical solution deposition (PACSD), which combines CSD and ultraviolet (UV) irradiation. At a low substrate temperature, the PACSD was able to form various oxide polycrystalline thin films, including SiO_2_ [147], TiO_2_ [146,148], Fe_2_O_3_ [149], SnO_2_ [150], ZnO [151], In_2_O_3_ [152], VO_2_ [153], and perovskites [154,155,156]. Epitaxial and highly oriented oxide thin films have also been prepared by improving the irradiation settings [157,158,159,160,161,162]. UV light is useful for the fabrication of oxide thin films since oxides often have high absorption, resulting in a shallow light penetration depth of less than 200 nm. One of the driving reasons for crystal development was explored as a photothermal effect under UV irradiation due to the high absorbance. Moreover, a photochemical effect in oxide film treatments has been described [163]. Controlling the crystal orientation and the preparation of various types of oxide thin films and film/substrate combinations are two methods that effectively leverage this phenomenon. In addition to pulsed UV laser processes, continuous UV lamps are expected to be a more cost-efficient process than pulsed lasers.

**Figure 14:**
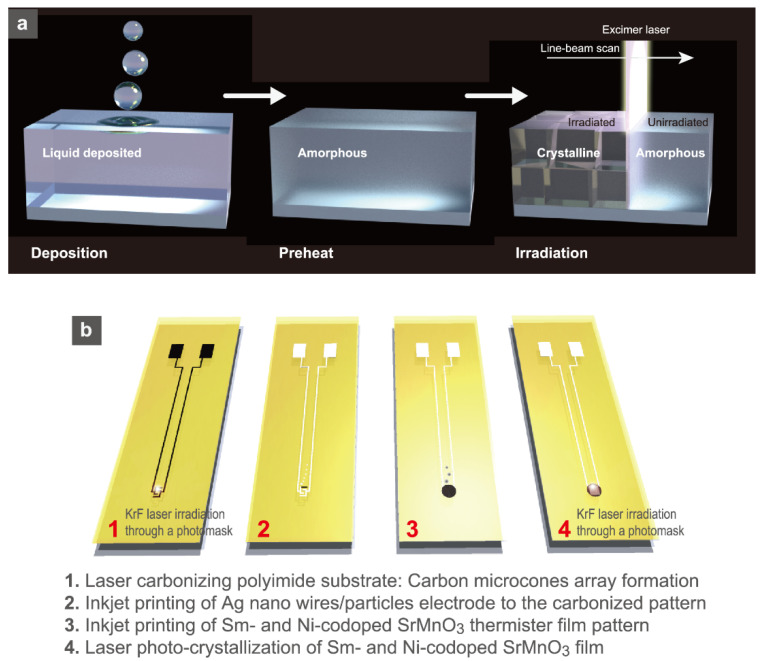
Schematics of (**a**) procedure of photocrystallization using an excimer laser for precursor films prepared by the CSD method. (**b**) Fabrication steps for Ag/carbon micro-cones composite electrodes and Sm- and Ni-codoped SrMnO_3_ thermistor sensors. Reprinted with permission from Ref. [53]. Copyright 2020 American Chemical Society.

**Figure 15:**
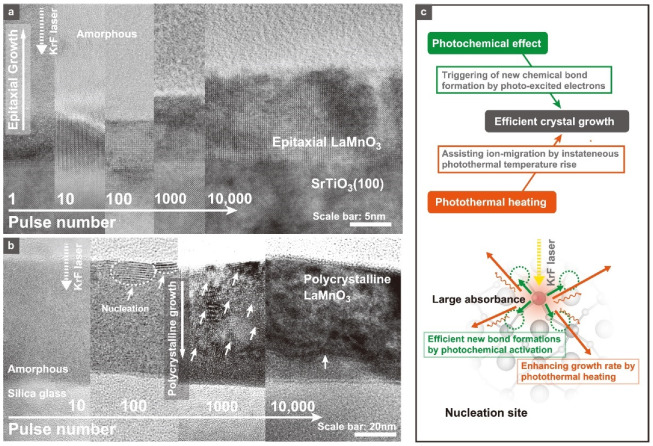
Cross-sectional transmittance electron microscope images for the LaMnO_3_ films on (**a**) SrTiO_3_(100) [163] and (**b**) silica glass substrates during KrF laser irradiation to a LaMnO_3_ amorphous matrix [164] by 10,000 pulse counts. (**c**) Proposed mechanism for the photocrystallization using pulsed UV lasers.

The PACSD can fabricate various oxide films at a very low substrate temperature by using pulsed UV lasers such as excimer lasers. The process is divided into three steps: solution deposition, preheating, and laser irradiation. Many oxides crystallize in air, and micro-patterning is straightforward since only irradiated parts are crystallized. As a result, this process is very suitable for fabricating compact sensor elements (Figure 14). The crystal nucleation and growth in the PACSD process using pulsed UV lasers are guided by the two fundamental features that are listed as follows. (1) In the absence of effective crystal nucleation sites in a film’s amorphous precursor matrix, crystal nucleation occurs first at the film surface along a gradient temperature profile to a depth of ~200 nm, as realized by excimer laser irradiation, owing to the amorphous precursor’s large photo-absorbance. Next, the crystal growth promptly progresses toward the substrate interface from the emerged crystal nuclei at the film surface (Figure 15a) [164,165]. (2) When a precursor matrix contains crystal nuclei with a high absorbance for the irradiated laser wavelength and a small lattice mismatch with the grown material, crystal growth proceeds rapidly from the effective nucleation sites due to photochemical activation for the formation of a new bond at the reaction interface (Figure 15b) [163].

When single-crystal substrates were used as effective nucleation sites in the PACSD process, for example, epitaxial growth was greatly enhanced even under small laser pulses compared to polycrystalline growth from the film surface [166]. In this case, the crystal growth proceeds preferentially from the substrate surface beyond the superiority of the gradient temperature distribution [157,161,162,163,167]. Figure 15c provides a quick summary. Based on these features, rapid polycrystalline growth can also be obtained by adding crystal nuclei into the starting precursor matrix, as first nucleation requires a large number of laser pulses. Certainly, when nanoparticles were put into the initial chemical solutions as target materials, rapid polycrystalline growth was confirmed in various experimental results. This can reduce the total number of pulses and also contribute to diminishing the damage to the substrate surface that has low heat resistance. The nanoparticle-containing dispersion inks are very well suited for low-temperature photocrystallization [53,168]. By using lamp light sources, such as excimer lamps [137,169,170] and flash lamps [58,171], instead of pulsed lasers, the flexible ceramic sensors can also be fabricated. Continuum light sources such as excimer lamps have the advantage of lower process costs, although the precursor compounds should be optimized to react at the specific wavelength of light since the peak power of light is commonly much lower than that of pulse light sources. As a result, the use of light has been employed as one significant technique for the fabrication of flexible ceramic sensors. Photocrystallization can only crystallize ceramics at specific sites that are directly irradiated by light sources, which is very useful for the surface functionalization of free-form sensor devices.

### 3.3. Transferring Methods

**Figure 16:**
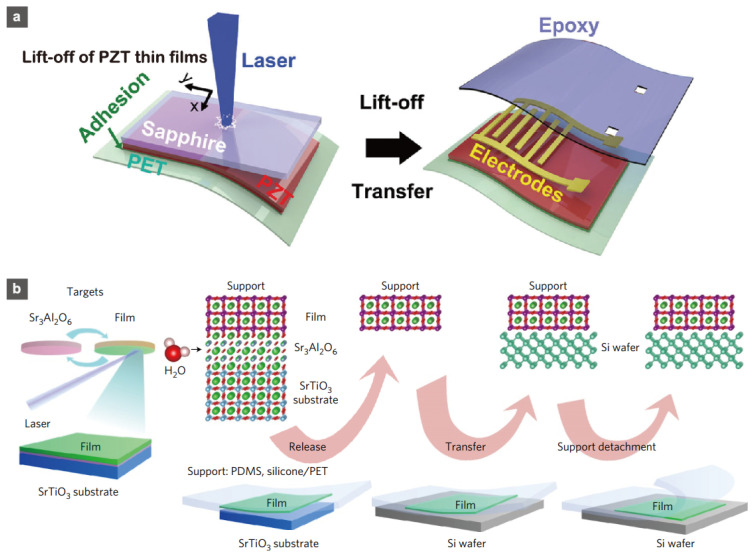
Schematics of the fabrication process for (**a**) a flexible, large-area PZT film by the laser lift-off method and (**b**) a flexible, large-area, epitaxial perovskite film by the etching of a sacrificial water-soluble layer method. Reprinted with permission from Refs. [142,172]. Copyright 2014 John Wiley and Sons and Copyright 2016 Springer Nature.

Transferring methods are also very strong tools for fabricating flexible ceramic films on plastic substrates. There are two commonly used transferring processes: (a) laser lift-off (LLO) [144,172,173]; and (b) the soluble sacrificial layer method [174,175,176,177] (Figure 16). Both processes first prepare high-quality ceramic films by conventional fabrication processes, such as physical vapor deposition on ceramic substrates, and then transfer the fabricated ceramic films to flexible substrates. According to Park et al., the LLO technique produces exceptionally high-quality PZT films on PET substrates [144]. They demonstrated large-area (1.5 cm × 1.5 cm) lift-off of PZT films from sapphire substrates onto PET. The PZT films that were created performed admirably as piezoelectric nanogenerators. Fabrications of high-quality, flexible, epitaxial ceramic films have been realized using the soluble sacrificial layer method. Nishikawa et al. and Lu et al. demonstrated epitaxial film forms for SrTiO_3_ thin films, using MgO and Sr_3_Al_2_O_6_ soluble sacrificial layers, respectively [174,175]. This method has the advantage of transferring high-quality films to target substrates in a non-destructive manner. These transferring processes are very useful for a wide range of materials and have strongly accelerated the fabrication of flexible ceramic films, including the aforementioned photocrystallization process.

## 4. Conclusions

Recent technological innovations such as material printing techniques and surface functionalization have strongly boosted the development of new free-form sensors for next-generation, flexible, wearable and 3D electronic devices. Ceramic film sensors, in particular, are in high demand for the production of reliable flexible devices. Various ceramic films can now be formed on plastic substrates through the developments in the fabrication process of ceramic films at a very low temperature, such as photocrystallization and transferring methods. Among the flexible sensors, strain sensors for precise motion detection, such as that of human muscles, and photodetectors for biomonitoring, such as for continuous glucose monitoring, have seen the greatest increases in research, while other fundamental sensors for temperature and humidity have also started to grow. Recently, flexible gas and electrochemical sensors have received a lot of attention from a new, real-time monitoring application that uses human breath and perspiration to accurately diagnose presymptomatic states. The realization of low-temperature processes for ceramic film sensors and their related components will complete the chemically stable and reliable free-form sensing devices by satisfying the demands that can only be addressed by flexible metal and organic components. Surface functionalization of ceramic film components on free-form substrates provides opportunities for new device design that is not dependent on shape of the electronic circuits. It will lead to the development of new applications, such as reliable skin patch biomonitoring sensor devices, 3D-structured advanced medical care instruments, and next-generation transport machines.

## Figures and Tables

**Table 1:** The *B* constant, substrate, and fabrication process for flexible thermistors that have recently been reported.

Material	*B* (K)	Substrate	Fabrication Process	Ref.
NiO	4337	Polyimide	Inkjet printing of nanoparticles and drying	[52]
NiO	8162	PET	Monolithic laser-induced reductive sintering	[46]
Ti_1−*x*_Al*_x_*N	2525	Polyimide	RF magnetron reactive sputtering	[51]
Mn_1.56_Co_0.96_Ni_0.48_O_4_	4429	PET	Photocrystallization of nanoparticles	[48]
Bi_4_Ti_3_O_12_	6515	Polyimide	Nanoparticle paste deposition and drying	[63]
Cs_2_SnI_6_	4400	Polyester	Inkjet printing of solutions and drying	[64]
amorphous-InGaZnO	2929	Metal foil	RF magnetron reactive sputtering	[47]
Sm_0.5_Sr_0.5_Mn_0.9_Ni_0.1_O_3_	2820	Polyimide (*t*: 5 μm)	Photocrystallization of nanoparticles	[53]
La_0.5_Ba_0.5_MnO_3_	2626	Al foil	Photocrystallization of nanoparticles	[65]

**Table 2:** The sensitivity, response time, and recovery time for inorganic humidity sensor materials.

Material	Sensitivity (%)	Response Time (s)	Recovery Time (s)	Ref.
WO_3−*x*_	276.8 (11–95%RH) ^r^	6	100	[71]
WS_2_	2357 (20–90%RH) ^r^	5 (35–40% RH)	6 (35–40%RH)	[72]
P (Black)	521 (32–97%RH) ^r^	101	26	[73]
MoSe_2_	625 (0–90%RH) ^c^	1.87	2.13	[74]
CeO_2_/g-C_3_N_4_	>700,000 (0–97%RH) ^c^	12 (0–43% RH)	—	[75]
V_2_O_5_	45.3 (11–97%RH) ^r^	240	300	[76]
Ni-doped Mn_3_O_4_	70 (11–44%RH) ^r^	120	141	[77]
SnO_2_	3200 (5–85%RH) ^r^	120–170	20–60	[78]
LiCl-TiO_2_	100,000 (11–95%RH) ^r^	3	7	[79]

The ^r^ and ^c^ represent resistive and capacitive, respectively. The sensitivity values are calculated from the resistance or capacitance variations at minimum and maximum %RH. The numbers in parentheses represent minimum and maximum %RHs.

**Table 3:** Gas-sensing properties and the morphology of nanoceramic flexible gas sensors.

Material	Morphology	Gas	Target Gas Concentration	Ref.
ZnO	Nanorods	Ethanol	10 ppm	[93]
ZnO	Nanowires	Oxygen	16 Torr	[94]
ZnO	Nanoparticles	Oxygen	200 ppm	[95]
ZnO	Nanoflowers	NO_2_	500 ppm	[96]
WO_3_	Nanowires	H_2_	500 ppm	[97]
WO_3_	Nanocolumnar	NO_2_	5 ppm	[98]
WO_3_·0.33H_2_O	Nanoneedles	Isopropanol	100 ppm	[99]
rGO/MoS_2_	Nanosheets	NO_2_	1.2 ppm	[100]
rGO/MoS_2_	Nanosheets	Formaldehyde	2.5 ppm	[101]
SnO_2_/Zn_2_SnO_4_	Nanoparticles	NH_3_	100 ppm	[102]
In_2_O_3_	Nanoparticles	H_2_S	100 ppb	[103]
In_2_O_3_	Nanoparticles	NH_3_	10 ppm	[104]
Amorphous-IGZO	Compact	NO_2_	5 ppm	[105]
TiO_2_	Nanopore network	Methanol	50 ppm	[58]
TiO_2_	Nanotubes	Trimethylamine	40 ppm	[106]
TiO_2_	Compact	H_2_	300 ppm	[107]
ZIF-7/TiO_2_	Nanoparticles	Formaldehyde	5 ppm	[107]
CuO	Compact	Acetone	0.8 ppm	[108]
SnS	Compact	NO_2_	5 ppm	[109]

## Data Availability

Not applicable.
